# Two novel *L2HGDH* mutations identified in a rare Chinese family with L-2-hydroxyglutaric aciduria

**DOI:** 10.1186/s12881-018-0675-9

**Published:** 2018-09-14

**Authors:** Wei Peng, Xiu-Wei Ma, Xiao Yang, Wan-Qiao Zhang, Lei Yan, Yong-Xia Wang, Xin Liu, Yan Wang, Zhi-Chun Feng

**Affiliations:** 1 Baiyi Children’s Hospital affiliated to PLA Army General Hospital, Beijing, 100700 People’s Republic of China; 2 National Engineering Laboratory for Birth Defects Prevention and Control of Key Technology, Beijing, 100700 People’s Republic of China; 3 Beijing Key Laboratory of Pediatric Organ Failure, Beijing, 100700 People’s Republic of China

**Keywords:** L-2-Hydroxyglutaric aciduria, *L2HGDH*, Mutation

## Abstract

**Background:**

L-2-Hydroxyglutaric aciduria (L-2-HGA) is a rare organic aciduria neurometabolic disease that is inherited as an autosomal recessive mode and have a variety of symptoms, such as psychomotor developmental retardation, epilepsy, cerebral symptoms as well as increased concentrations of 2-hydroxyglutarate (2-HG) in the plasma, urine and cerebrospinal fluid. The causative gene of L-2-HGA is L-2-hydroxyglutarate dehydrogenase gene (*L2HGDH*), which consists of 10 exons.

**Case presentation:**

We presented a rare patient primary diagnosis of L-2-HGA based on the clinical symptoms, magnetic resonance imaging (MRI), and gas chromatography-mass spectrometry (GC-MS) results. Mutational analysis of the *L2HGDH* gene was performed on the L-2-HGA patient and his parents, which revealed two novel mutations in exon 3: a homozygous missense mutation (c.407 A > G, p.K136R) in both the maternal and paternal allele, and a heterozygous frameshift mutation [c.407 A > G, c.408 del G], (p.K136SfsX3) in the paternal allele. The mutation site p.K136R of the protein was located in the pocket of the FAD/NAD(P)-binding domain and predicted to be pathogenic.

**Conclusion:**

We predicted the homozygous missense mutation (c.407 A > G, p.K136R) was considered as the pathogenic mutation of the patient. The study highlights the power of pedigree analysis in order to interpret novel mutations.

## Background

L-2-Hydroxyglutaric aciduria (L-2-HGA; OMIM 236792) is a rare organic aciduria that was first described by Duran in 1980 [1]; this neurometabolic disease is inherited as an autosomal recessive mode. Approximately 300 cases have been reported worldwide (as of 2014) [2]. The diagnosis of L-2-HGA can be made based on clinical evaluation, magnetic resonance imaging (MRI), urinary organic acid screening by gas chromatography-mass spectrometry (GC-MS), and mutational analysis of L-2-hydroxyglutarate dehydrogenase (*L2HGDH*) gene. The main clinical manifestations of the disease include psychomotor developmental retardation, epilepsy, and cerebral symptoms. It usually has an insidious onset during the first year and progresses slowly. The disease does not present an acute deterioration course that finally leads to a delayed clinical diagnosis in childhood, so most patients can live to adulthood. The characteristics of MRI are lesions in the subcortical white matter, with cerebellar atrophy and abnormal signals in the dentate nuclei and putamens. L-2-HGA is characterized with increased concentrations of 2-hydroxyglutarate (2-HG) in body fluid, such as plasma, urine and cerebrospinal fluid. The causative gene of L-2-HGA is located on chromosome 14q22.1, which is called the *L2HGDH* gene and consists of 10 exons. Topcu [3] first identified the gene using homozygosity mapping and reported nine mutations in 15 families. The gene possibly encodes L2HGDH, which is a flavin adenine dinucleotide (FAD) -dependent, membrane-bound dehydrogenase, is located in mitochondria, and catalyzes L-2-hydroxyglutarate metabolizing to α-ketoglutarate. L2HGDH plays a role in metabolite repair by eliminating the useless metabolite in the body [4].

Reports about patients with L-2-HGA in Chinese people are rare. Here we reported two novel mutations in the *L2HGDH* gene, which was detected in a rare Chinese boy with L-2-HGA. We also evaluated his family pedigree.

## Case presentation

A 5-month-old boy presented with psychomotor developmental retardation and was admitted to the pediatric neurodevelopmental unit of Bayi Children’s Hospital. He could not control his head, was prone to hypsokinesis, was unable to turn over or grasp, and had high muscle tension. The parents of the patient denied heredity history or consanguineous marriage.

A urine sample was prepared with urease pretreatment methods, according to the method in the reported literature [5]. Derivatization was performed with 100 μL bis-(trimethylsilyl) trifluoracetamide (BSTFA) + 1% trimethylchlorosilane at 90 °C for 40 min, the metabolites were analyzed by GC-MS after cooling. The final derivatized metabolites were injected into the GC-MS, using a split-less mode per microliter. The temperature of the injection port was set at 250 °C. The initial temperature of the column oven was 60 °C and held for 2 min, then increased with the speed of 10 °C/min until reaching the temperature of 220 °C, held for 3 min; then it was increased with the speed of 15 °C/min to the final temperature of 325 °C and held for 5 min. The temperature of the ion source and transfer line was 280 °C and 300 °C, respectively. The run time lasted for 33 min, and the scan range was programmed from m/z 50 to 550. Finally, qualitative analyses of the characteristic mass spectrogram of each flow peak and the ratio of the peak area of 2-HG to that of creatinine was used as the quantitative index [6].

Following the manufacturer’s instructions, genomic DNA was isolated from the peripheral blood specimens of the three people (patient and his parents) with the RelaxGene Blood DNA System (TianGen, Beijing, China). PCR amplified the entire coding region sequences, covering all 10 exons and the exon-intron boundary sequences of *L2HGDH* with primers as described by Topcu [3]. The PCR products were sequenced directly on an ABI 3730 sequencer (Applied Biosystem, Foster City, USA). The results were analyzed with the Gene tool program and Chromas program. The naming of the mutations was on the basis of Human Genome Variation Society (HGVS) guidelines (http://www.hgvs.org), according to GenBank NCBI reference sequence NM_024884.2. We used three prediction software programs, SIFT, PloyPhen-2, and MutationTaster, to predict the potential pathogenicity of the sequence mutations. On structural analysis, the protein (Uniport ID: Q9H9P8) of L2HGDH for human was downloaded from Uniport (https://www.uniprot.org/). Then its three-dimensional structure was predicted using I-TASSER (https://zhanglab.ccmb.med.umich.edu/I-TASSER/) with the default parameters and the model with the highest C-score was selected. We used the InterPro (https://www.ebi.ac.uk/interpro/) to predict the domain of protein Q9H9P8. Finally, the predicted structure and the functional domains were visualized by PyMol 2.1 (https://pymol.org/2/).

**Fig. 1:**
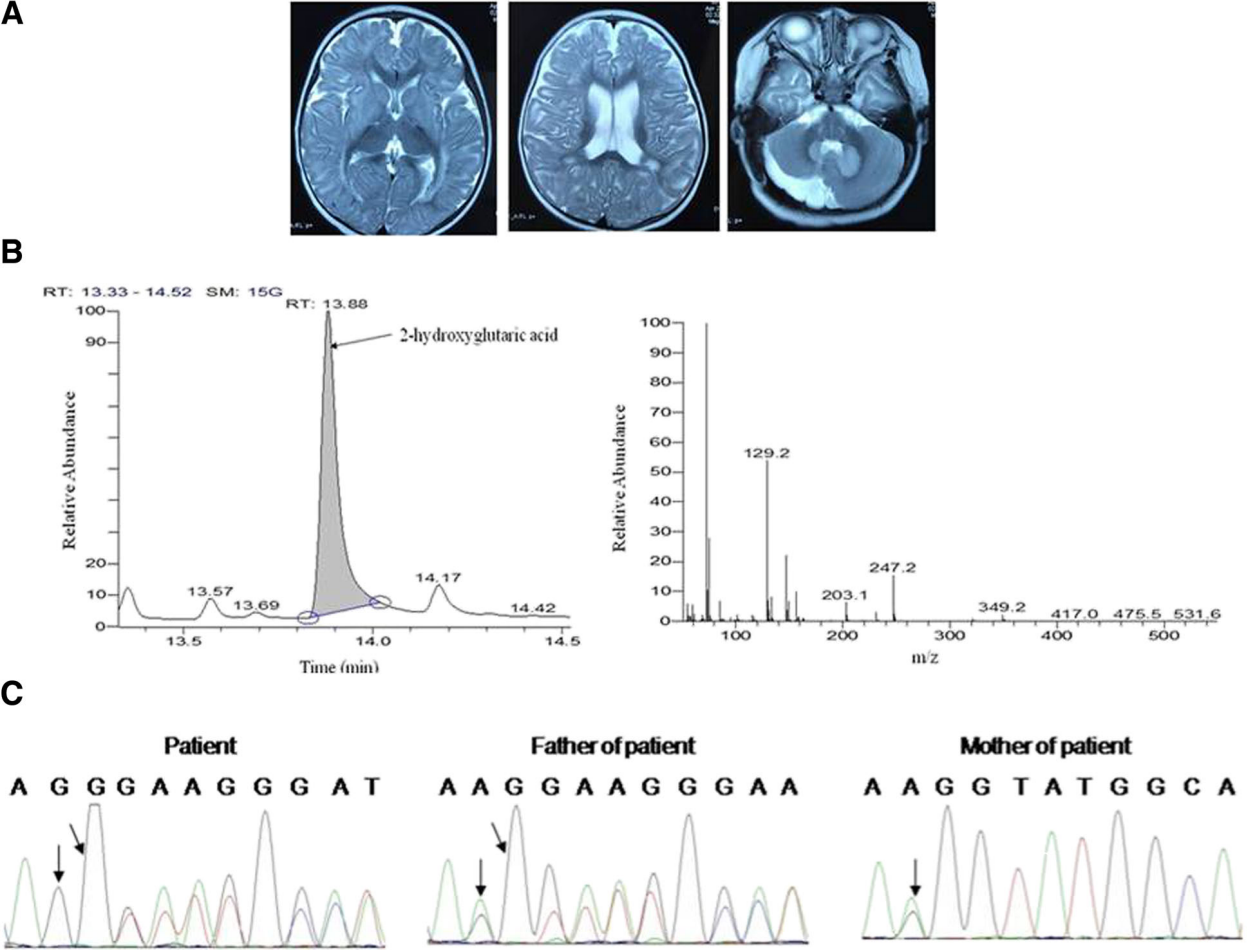
The brain magnetic resonance image (MRI) results of the patient (**a**). T2-weighted image revealed symmetrical, high-signal changes in the subcortical white matter, basal ganglia, and cerebellar dentate nuclei. The lateral ventricular wall showed multiple nodular gray matter heterotopia. The right side of the cerebellar hemisphere and vermis showed dysplasia. Total ion chromatograms (TIC) and the mass spectrum of the patient (**b**). Results of *L2HGDH* mutational analysis (**c**). Arrows indicate mutation sites. The patient had two novel mutations, one of which was a homozygous missense mutation (c.407A > G, p.K136R). The other one was a heterozygous deletion (c.408delG, p.K136SfsX3). His father showed both the heterozygous missense mutation and the heterozygous delete mutation. His mother had the heterozygous missense mutation (c.407 A > G, p.K136R)

Brain MRI revealed symmetrical, high-signal changes in the subcortical white matter, basal ganglia, and cerebellar dentate nuclei. The lateral ventricular wall showed multiple nodular gray matter heterotopia. The right side of the cerebellar hemisphere and vermis showed dysplasia (Fig. 1a).

GC-MS analysis showed visibly increased excretion of 2-HG (209.70 mmol/mol cre, normal range 0.00~ 19.85 mmol/mol cre) in the urine. The peak area ratio of 2-HG was 20.84, and the normal peak area ratio was 0.25, which was the 99.5 percentile of 500 newborns with normal metabolism. The retention time was 13.88. The characteristic ion used for quantification was m/z 349 (Fig. 1b).

**Fig. 2:**
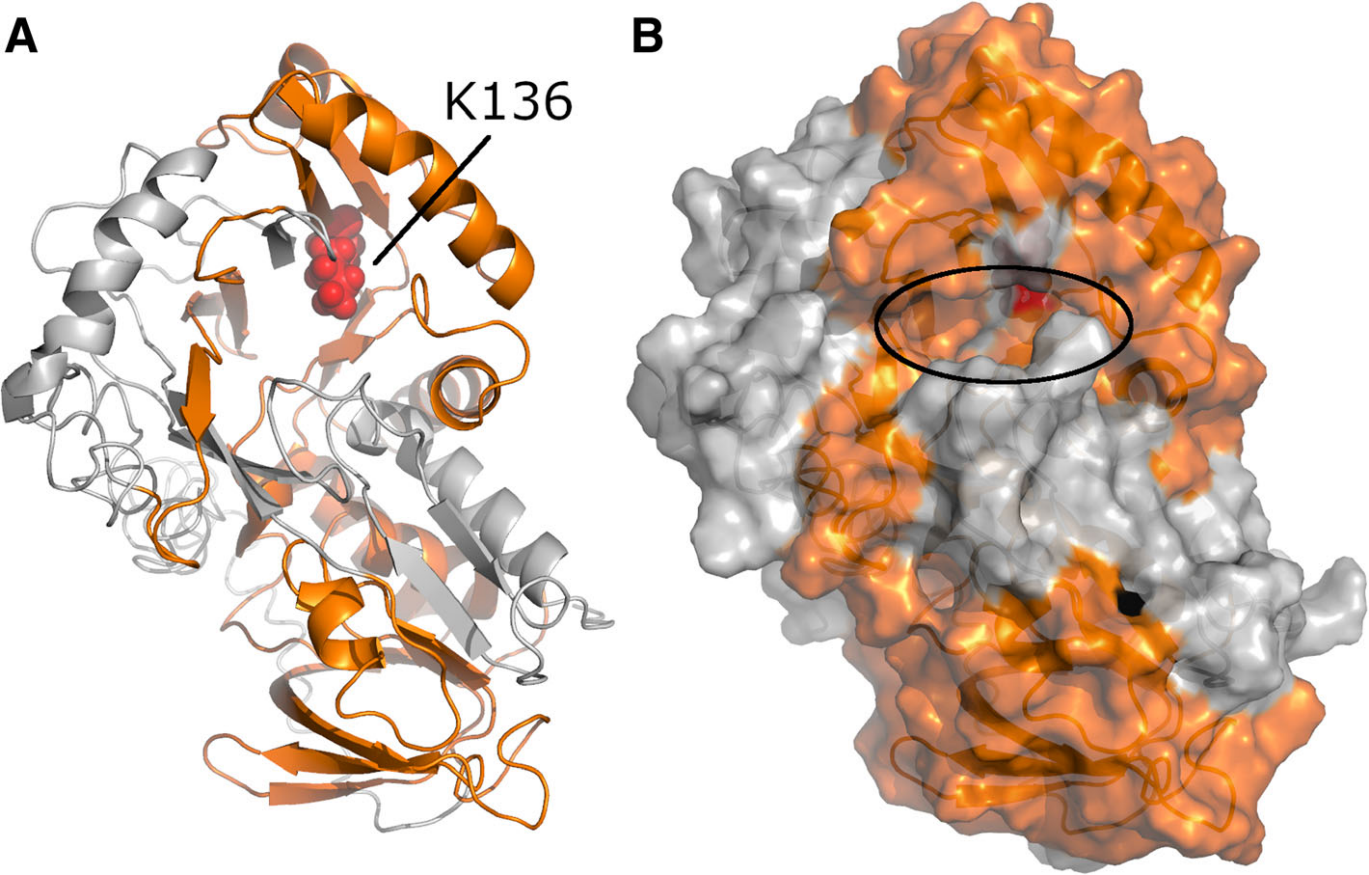
The predicted structure of the protein of L2HGDH in cartoon (**a**) and surface mode (**b**). The FAD/NAD(P)-binding domain was highlighted as orange. The mutation site 136 of the protein was shown in red spheres which was located in the pocket (shown in th black ellipse) of the FAD/NAD(P)-binding domain

We identified two novel mutations in exon 3 of the patient. One was a homozygous missense mutation (c.407 A > G, p.K136R). We used three prediction software programs, SIFT, PloyPhen-2, and MutationTaster, to predict the potential pathogenicity of the sequence mutations. The results were “damaging (0 score)”, “probably damaging (1 score)” and “disease causing (26 score)” respectively. This mutation was not present in the 1000 Genome Project, NHLBI GO Exome Sequencing Project and and InNormal Database (MyGenostics, number of 1000). The mutation site p.K136R of the protein was located in the pocket (shown in th black ellipse) of the FAD/NAD(P)-binding domain(Fig. 2). The other mutation was a heterozygous deletion of one nucleotide (c.408delG, p.K136SfsX3), which would be expected to cause a premature stop codon at position 138 in the protein sequence. Further pedigree analysis of the healthy parents confirmed that the two novel mutations segregated with the disease in an autosomal recessive trait. His asymptomatic mother had the heterozygous missense mutation (c.407 A > G, p.K136R). His asymptomatic father showed both the heterozygous missense mutation (c.407 A > G, p.K136R) and the heterozygous delete mutation (c.408 del G, p.K136SfsX3) (Fig. 1c). Finally, we predicted the homozygous missense mutation (c.407 A > G, p.K136R) was the pathogenic mutation of the patient.

## Discussion and conclusions

In our study, we describe the clinical manifestation, MRI, GC-MS, and genetic findings in a rare patient with L-2-HGA. Our study also highlights the power of pedigree evaluation to interpret novel sequence variants.

The clinical manifestations of L-2-HGA are homogeneous, including insidious onset, developmental delay, epilepsy, and cerebellar ataxia [7–9]. The age of onset with L-2-HGA reported in literature ranges from infancy to 35 years [10]. The patient in our study had symptom onset at 5 months of age, with loss of milestones and developmental delay. Characteristic MRI in L-2-HGA demonstrates predominant subcortical cerebral white matter and dentate nucleus abnormalities, globus pallidus, putamen, and caudate nucleus. The brain MRI of our patient showed cerebral white-matter and cerebellar dentate nuclei abnormalities consistent with the diagnosis and was special in multiple nodular gray matter heterotopia. Urinary organic acid screening by GC-MS showed visibly increased excretion of 2-HG, but the chiral configuration was not determined. Although the clinical presentation and MRI findings can help to determine either D-2-Hydroxyglutaric aciduria (D-2-HGA) or L-2-HGA, the differentiation of chiral configuration is necessary for the right differential diagnosis. 2-Hydroxyglutaric aciduria (2-HGA) is divided into three types, including L-2-HGA, D-2-HGA, and D, L-2-HGA, according to the chital configuration of 2-HG. The age at onset and predominant developmental delay with the classical MRI findings suggest the diagnosis of L-2-HGA in our patient.

Genetic analysis of the causative gene, *L2HGDH*, can further confirm L-2-HGA and aid in genetic counseling and future prenatal diagnosis. We analyzed the sequence of *L2HGDH* in our patient. Thus far, over 110 variants throughout the *L2HGDH* gene have been reported in the Leiden Open Variation Database (http://www.LOVD.nl/). Among these variants, missense mutations are the most frequent changes (approximately 32%); nonsense mutations, splicing site mutations and frameshift mutations are at a proportion of 9% to 12%. The *L2HGDH* gene is reported as an autosomal recessive model, and it is possible that homozygous or compound heterozygous mutations in two chromosomes could cause L-2-HGA. A homozygous missense mutation and a heterozygous, one-nucleotide deletion mutation were detected in our patient. The two novel mutations were both located in conserved sequences. The frameshift mutation severely disrupted the coding of the protein, which was called loss-of-function. It is traditionally considered as more deleterious [11]. Therefore, the frameshift was first expected to be pathogenic. Furthermore, we conducted the pedigree evaluation and detected that the homozygous missense mutation came from his father and mother, and the frameshift mutation came from his father. We speculated that these two mutations of his father located on the same allele and the other allele compensated for the normal function, so his father was asymptomatic. We did not find the missense mutation in 100 control chromosomes in the Chinese cohort, the amino acids are known to be highly conserved. The structural analysis of the protein was investigated through I-TASSER, InterPro and PyMol 2.1. The mutation site p.K136R of the protein was located in the pocket of the FAD/NAD(P)-binding domain. A study of Muhammad Ikram [12] showed a novel homozygous mutation in *L2HGDH* gene (c.178 G > A, p.G60R). The mutation p.G60R lies in the same highly conserved FAD/NAD(P)-binding domain, predicted to disturb enzymatic function. And we predicted the mutation p.K136R to affect enzymatic function. There is little information about the structural function annotation of this protein and the function of the pocket have not been found in literature before, We need more in-depth research on the protein. Moreover, the frameshift mutation was behind the missense mutation which had already changed the function of the gene. Above all, we predicted the homozygous missense mutation (c.407 A > G) was considered as the pathogenic mutation of the patient which resulting in the replacement of lysine by arginine (p.K136R).

The disease also has an increasing morbidity of cerebral tumors, of which the prevalence would be approximately 5% [8, 13]. In our study, the patient did not present with cerebral neoplasms perhaps because it was too early to occur; the neoplasms usually occur between the age of 3 and 36 years of L-2-HGA patients [8]. A study reported the mechanism of tumorigenesis between L-2-HGA and brain tumors in isocitrate dehydrogenase 1 (*IDH1)* gene mutations [10, 11, 13].

In summary, this study provides a good example that we should do our best to interpret the novel sequence variants. Whenever possible, variants of uncertain significance should be further investigated with pedigree analysis before excluding pathogenicity.

## Abbreviations

2-HG: 2-hydroxyglutarate
BSTFA: Bis-(trimethylsilyl) trifluoracetamide
D-2-HGA: D-2-Hydroxyglutaric aciduria
FAD: Flavin adenine dinucleotide
GC-MS: Gas chromatography-mass spectrometry
IDH1: Isocitrate dehydrogenase 1
L-2-HGA: L-2-Hydroxyglutaric aciduria
L2HGDH: L-2-hydroxyglutarate dehydrogenase
MRI: magnetic resonance imaging
